# “Death is inevitable – a bad death is not” report from an international workshop

**DOI:** 10.1186/s13584-019-0348-y

**Published:** 2019-11-12

**Authors:** Adir Shaulov, Kassim Baddarni, Nathan Cherny, Dorith Shaham, Pesach Shvartzman, Rotem Tellem, A. Mark Clarfield

**Affiliations:** 1 Department of Hematology, Hadassah-Hebrew University Medical center, Jerusalem, Israel; 2 Al-Taj for Health and Heritage Organization, Arraba, Israel; 3 Department of Palliative care, Shaarei Zedek Medical Center, Jerusalem, Israel; 4 Department of Radiology, Hadassah-Hebrew University Medical center, Jerusalem, Israel; 5 Department of Family Medicine and Palliative Care Unit, Clalit Health Services and Siaal Research Center for Family Medicine and Primary Care, Ben-Gurion University of the Negev, Beer-Sheva, Israel; 6 Palliative Care Service, Tel Aviv Medical Center and Sackler School of Medicine, Tel Aviv University, Tel Aviv, Israel; 7 Department of Geriatrics, Soroka Medical center, Beer Sheva and Faculty of Health Sciences, Ben-Gurion University of the Negev, Beer-Sheva, Israel

**Keywords:** Palliative care, Hospice, Spiritual care, Truth telling, Death and dying

## Abstract

Palliative care is an approach meant to improve the quality of life of patients facing life-threatening illness and to support their families. An international workshop on palliative care took place in Caesarea, Israel under the auspices of the National Institute for Health Policy Research on July 4-5th, 2018, with the goal of discussing challenges to the development and integration of palliative care services in Israel. At the workshop, both national and international figures in the field of palliative care and health policy addressed several issues, including truth telling, religious approaches to end of life care, palliative care in the community, pediatric palliative care, Israel’s Dying Patient Act, the Ministry of Health’s National Plan for palliative care, and challenges in using advance directives. We summarize the topics addressed, challenges highlighted, and directions for further advancement of palliative care in the future, emphasizing the critical role of the Ministry of Health in providing a framework for development of palliative care.

## Introduction

Palliative care (PC) is an approach dedicated to the improvement of quality of life for patients suffering from life threatening diseases and support for families, through prevention and relief of physical, psychosocial and spiritual suffering [1, 2]. PC has been recognized to relieve pain and suffering, improve quality of life, reduce medical expenditures, and even prolong life [1, 2]. As such, it has become the standard of care for patients facing incurable malignancy and other life limiting illnesses [3, 4].

In Israel, the Dying Patient Act was enacted in 2005 and the Ministry of Health (MoH) defined the populations for which PC should be made available in 2009. However, despite these measures, rudimentary PC services are currently provided only to a small population of mostly cancer patients [5, 6]. Only two inpatient hospices exist in Israel, in Tel HaShomer and Hadassah Mount Scopus hospitals, established in 1983 and 1986 respectively. These services continue to exist, but under a constant threat of closure due to insufficient remuneration for admissions [7]. In many geriatric facilities where many of the country’s deaths occur, staff often lack the skills and knowledge needed for quality palliative care and have limited access to specialized palliative care services. Access to opioids and other palliative medications in these facilities is limited while regulatory pressures [8] and cultural norms [9] result in a high rate of tube feeding. While preliminary homecare services were jumpstarted by the Israeli Cancer Association in the early 1980s, [5] it was not until 2009 that the MoH mandated access to PC services for all, [10] thus creating a groundswell of home palliative care services run by the health funds or contracted by them to private companies. While there has been remarkable growth in home services, staff training and competence remains variable and availability of these services for non-cancer populations remains limited [11]. Despite a clear need for inpatient palliative care services, until 2015 few such services existed [12, 13]. In 2015, the MoH conducted a survey of hospital and institutional preparedness for end-of -life treatment [14]. In effort to meet the regulatory criteria, many hospitals and geriatric institutions created palliative care teams to address these issues and developed local practices and procedures. However, as specific ring-fenced funding and training were not provided for these programs, many palliative care teams are understaffed and untrained, thus drastically affecting the quality of care provided by these teams.

Training of PC nurse specialists began in 2009 [15] and PC was formally recognized in 2013 as a medical specialty in Israel. Still, fellowship and specialist positions are rare, due primarily to lack of funding. PC training programs and have been developed [16–18], however the vast majority of clinicians have had no training in PC at either an undergraduate or postgraduate level.

In a few institutions, spiritual care programs have also developed and these seem to be well received [19]. The Association for Spiritual Care in Israel was established with the support of the UJA-Federation of New York in 2015. Although spiritual care in Israel has made progress in standardization and accreditation, funding constraints and a misconception associating spiritual care with religion have proven to deter both patients and providers [20, 21].

A national plan for PC addressing many of these issues has been provided by the MoH, and implementation of its recommendations has begun [22].

In order to address the challenges of and goals for integration of PC in Israel into health care, an international workshop was held in Caesarea on July 4-5th, 2018. The conference was organized and funded by the Israeli National Institute for Health Policy Research (NIHP), a governmental organization which periodically hosts conclaves on major health care policy issues in Israel.

The conference exposed attendees to the experience of established PC systems from the United States, Canada, and the United Kingdom, in addition to highlighting areas of expertise and experience within Israel.

Major topics addressed included:

- A review of the history and practice of palliative care
- Truth telling
- Religious and spiritual approaches to end-of-life (EOL)
- PC in the community
- Pediatric PC
- Israel’s Dying Patient Act – the law and practice
- The National Plan for Palliative and EOL care
- The challenge of advance directives

### Openings

Prof. Orly Manor, chair of the board of NIHP, opened the conference, followed by Prof. A. Mark Clarfield, scientific chair, who introduced the major themes of the meeting and its main goal: to learn from one another in order to further the integration of PC into Israel’s health care services. He thanked the NIHP for its support and particularly expressed his gratitude to fellow members of the scientific organizing committee for their guidance and help throughout the process of planning the workshop.

Baroness Prof. Ilora Finlay, of the UK, opened the introductory session via videoconference. She elaborated on the history of PC and provided an international view of practice today. She recounted that the clinical field was introduced by Dame Dr. Cicely Saunders in 1967, ushering in the dawn of the hospice movement which emphasized quality EOL care. This development was followed by an increasing understanding of the value of early PC and its benefits for quality of life throughout the course of disease. In parallel with judicious treatment of the underlying condition to avoid harm, PC can also prolong life.

Dr. Gil Siegal from Israel outlined the various stakeholders in PC, beginning with patients, families, and caregivers, as well as the medical system and medical institutions. Siegal then discussed the involvement of social and governmental institutions, by regulating the withdrawal and withholding of treatment and access to PC. Interacting and conflicting interests and values such as sanctity of life, dignity, the balance between beneficence, (aka “paternalism”) and autonomy, along with communal ideals all have a unique expression in Israel. All these are expressed in legislation and social constructs surrounding the end of life.

Dr. Daniel Sulmasy from the US delivered a presentation on the value of “spirituality”, defined as one’s relationship with the transcendent questions that confront us as human beings, in PC. This notion contrasts with “religion,” which he defined as a set of texts, practices, and beliefs about the transcendent, shared by a particular community. While many patients want their physician to inquire about their spiritual needs, this rarely happens [23]. Dr. Sulmasy offered that clinicians hesitate to address spirituality for fear of invading privacy as well as difficulty in facing the limits of medicine. However, not addressing spiritual needs is a strong predictor of dissatisfaction with and low quality of care [23, 24].

The major questions addressed by spiritual care include *meaning* (hope and despair), *value* (dignity and indignity) and *relationships* (reconciliation and alienation). Dr. Sulmasy also touched on specific beliefs about death and dying in various religions (see below for further elaboration). He urged clinicians to attend not only to the patient’s spirituality, but even—if possible—to their own.

### Group work I

Participants were split into three groups (12–16 people each), to discuss a clinical case of a young ultra-orthodox Jewish man with terminal lymphoma; his father managed his treatment and refused to allow the administration of opiates for pain and dyspnea at EOL (see appendix). Relevant issues were discussed from ethical, legal, religious, and cultural perspectives. The challenges of administering PC in complex social, cultural, and religious settings were addressed.

### To tell or not

The next session highlighted the issue of truth telling in clinical care. Prof. Ora Paltiel outlined her personal experience and difficulty transferring her Canadian training in truth telling to a different cultural setting in Israel after moving there in the middle of her career [25]. Although many believe that truth rests at the top of the hierarchy of values, there are many reasons—with varying degrees of justification—not to disclose complete information. These include health practitioner discomfort, concern for negative impact on patients, true uncertainty about prognosis and illness trajectory, lack of time, requests from patients, and specific, often forceful, requests from family to withhold information [26, 27]. While Israel’s Patient’s Rights Law now dictates that a physician is obligated to convey the necessary medical information that a ‘reasonable’ patient would require in order to make an informed decision, the legislation provides an exception (at least in theory), should a Hospital Ethics Committee establish that providing particular information might seriously harm the physical or mental health of the patient. It turns out, however, that this mechanism is very infrequently used.

Over past decades, truth telling and patient autonomy have become near moral absolutes in most western societies, while in other, more family-centric settings, personal autonomy may not be the foremost ethical principle wished for by patients or their families [28, 29]. In Japan, for example, family-centered truth telling has been the norm, in contrast with the US or Canada, where patients usually value a more individualized approach [29].

Models of truth telling include partial or complete nondisclosure, (citing the ethical principle of beneficence), full disclosure, (emphasizing autonomy), and individualized disclosure (emphasizing a more nuanced, patient-centered, and guided communication) [30]. To this end, truth “offering” has been suggested as a path to individualize the transmission of clinical information and prognosis in PC, allowing patients leeway in deciding the level of information they would actually wish to be exposed [31]. Most workshop participants agreed that cultural competence, awareness of diversity, and most importantly, cultural humility (a process that brings into check the inherent imbalance of the physician-patient communication), should be included in the medical curriculum [32].

In discussing Prof. Paltiel’s paper, Prof. Nathan Cherny, also from Israel, offered a theoretical framework clarifying that disclosure of diagnosis, extent and seriousness of disease, and therapeutic options is critical for informed decision making. Although common understanding is that disclosure is essential for preserving autonomy, he agreed with Prof. Paltiel that a more nuanced approach might often be preferred. Voluntarily diminished autonomy may be expressed by either delegating this autonomy to someone else or requesting diluted disclosure of potentially distressing information. Relational autonomy may also take into account the varying obligations, duties, and ideas of reciprocity and interdependence held by all parties involved, factors which are all affected by cultural values. As Prof. Paltiel had pointed out, cultures often differ in value orientations such as egalitarianism, harmony, embeddedness, hierarchy, mastery, affective autonomy, and intellectual autonomy [33]. Culture can also influence patient expectations of truth telling in many ways, including patient assumptions and their attitudes toward risk-taking behavior and non-confrontation.

As the poem goes, “No man is an island.” Beyond the patient, family members’ demands and expectations can be similarly affected by culture. Furthermore, professional norms and expectations for full disclosure or precautionary paternalism may vary across cultures. ‘Hard’ paternalism via precautionary intervention may sometimes be justified in order to protect genuine patient interests within the asymmetry of knowledge and judgement present in the medical setting. The practice of truth telling should preferably be done in the manner that is least restrictive to patient autonomy, using titrated or partial disclosure.

The current consensus supports a preemptive clarification of information preferences. At times, the family is informed of a patient’s diagnosis first, due to presumed consent, convenience, paternalism, or local norms. In such cases, family requests for non-disclosure may result not from cultural differences but rather from anxiety, a lack of understanding of the clinical situation, a desire for control, a genuine wish to reduce harm to the patient, or all of these in some combination. As a last resort, in the face of an intractable moral conflict, the clinician may choose principled non-cooperation with such a request from family members or participation under protest.

### Group work II

A second small group discussion (see appendix) addressed the issue of tube feeding in an elderly patient with severe neurological deficit; the family had requested to deny information to the patient’s spouse when he queried the medical team daily about her prognosis. The groups discussed limitations of the law in cases where there are no advance directives (AD) and no recognized surrogate for decision-making. In addition, participants analyzed requests to deny information to both patient and spouse.

### Role of religion: Islam, Catholicism, and Judaism

Mr. Kassim Baddarni presented the approach of Islam, wherein the human body is seen as entrusted to man but ultimately belonging to Allah; as a result, it is deemed sacred. Islam also holds that all creatures will die, but only Allah may decide when, where, and how. Muslims still feel a religious duty to prevent and treat illness and to preserve and prolong life insofar as is possible*.* Like Judaism, but in contrast to Catholicism, *Sharia* (formal Islamic law) forbids euthanasia, suicide, and assisted suicide. Forced feeding of the sick and exposing oneself to health hazards are also discouraged.

With respect to life support, if three knowledgeable physicians agree that the patient’s condition is hopeless, artificial life support can be withheld or (in contrast with Judaism) even withdrawn. However, again echoing Judaism but contrasting with Catholicism, basic nutrition, hydration, nursing, and relief of pain are all considered “ordinary” care and must not be withheld. While pain and suffering are seen as a test from God, they are not meant as punishment. The Muslim perspective on a good death includes dignity and privacy, provision of spiritual and emotional support, access to hospice care, pain and symptom control, ability to issue advance directives, time to say goodbye, the ability to leave when it is one’s time to go, and finally, the ability to retain control [34].

According to Islam, upon death, all of a person’s deeds come to an end except for three: perpetual charity, knowledge which is beneficial, and a virtuous descendant who prays for him. As is also the case in Judaism but not Catholicism, burial must be arranged as quickly as possible after death. The burial process consists of reciting a special prayer (*Salat Janaza*), washing the body three times (*Ghusl*), preparing it by closing the mouth and eyes and straightening the arms and legs, and finally shrouding the body (*Kafan*). The body is positioned on its right side and is buried without a coffin, facing Mecca.

Dying well in Islam has religious connotations – it signifies dying at peace with God. Medical concerns include being kept comfortable and free of pain. Community connotations include having added value to the lives of others before death.

All participants concurred that an understanding of culture and tradition can improve care, avoid misunderstandings, and reduce the chance of resultant conflict between staff and family [35].

Dr. Daniel Sulmasy presented the ethics of EOL care from a Catholic perspective. In Catholicism, while life is inherently valuable and considered a gift from God, only God is infinitely of value. Therefore, while people have a duty to care for themselves, that obligation is limited, and interventions can be withheld or withdrawn when they are judged futile or when the burden of care outweighs the benefits. While euthanasia and assisted suicide are always considered morally wrong, morphine and similar drugs can be given to dying patients, in accordance with the principle of double effect [36]. While withholding and withdrawing care are not seen as intrinsically different in Catholicism, it was acknowledged by almost all participants that, from a psychological point of view, withdrawing is always a more difficult action.

Prof. Shimon Glick then addressed orthodox Judaism’s formal approach to EOL care, which is strongly pro-life. This view is best expressed by the Talmudic lesson, “Therefore was Adam created as a single individual – to teach us that one who saves a single life is as if he saved an entire world, [and he who destroys a single life is as if he has destroyed an entire world]”. This emphasis provides a powerful imperative for *pikuach nefesh*, the obligation to save a human life, which overrides all the commandments in the Jewish religion, save three (defamation of God’s name, forbidden sexual relations, and murder).

While active euthanasia and suicide are absolutely forbidden, under specific circumstances of irremediable suffering and incurable illness, one is not required to provide *active* treatment. In general, suffering is not viewed positively, and active treatment of distress is mandated, even at the potential risk of shortening life—similar in intent to the principle of double effect allowed by Catholicism. In contrast to prevailing norms in Western bioethics, orthodox Judaic tradition does differentiate between the withholding and withdrawal of therapy, with most authorities in orthodox Judaism allowing the former while forbidding the latter. An interesting discussion followed, comparing and contrasting the Jewish, Christian and Muslim approaches to EOL care [37].

### PC in the community

The second day of the conference opened with **Dr. Sandy Buchman,** the president-elect of the Canadian Medical Association and a well-known specialist in PC. He described the essential components of a well-functioning and sustainable PC system. Dr. Buchman emphasized the necessity for broad, integrated services which are accessible, equitable, and responsive to the needs of patients and families across a continuum of care, as these needs may evolve over time. Challenges to this ideal include inequity of access to services, which is often seen in non-cancer patients and in pediatrics, [38] as well as providing community-based PC, in alignment with the popular preference to die at home [39].

For various reasons, many patients do not access PC services, and nearly half of patients who receive such care do so only in their last month of life. Buchman held that PC should be integrated with primary care practitioners, who can identify patients in need of such terminal care earlier, provide effective primary palliative care, and provide a continuum of care in various settings over time. Improved training, mentorship, and support by specialized PC teams via regional networks may enable growth of PC capacity and provision of care throughout the continuum of care. Dr. Yoram Zinger responded to Dr. Buchman’s presentation by addressing the limited availability of PC services in Israel today.

### Dying children: a different challenge

A panel discussion on pediatric palliative care (PPC) was opened by Prof. Pesach Shvartzman, who described the approach to the most common diagnoses children face: genetic syndromes, congenital abnormalities, neuromuscular disease, and cancer. Mrs. Yael Ben Gal elaborated on the unique nature of a child’s death –usually experienced as an occurrence against nature, a veritable failure, and always a tragedy – which makes it more difficult to accept and leads to a prolonged and difficult grieving process for parents. In PPC, what is best for the child may not always align with the parents’ perceived needs. This discord can play a major role in decision-making. In adolescence, although the patient has not reached the “legal” age of decision, issues of understanding the implications of an illness and of decision-making become more prominent and difficult.

Dr. Sergey Postovsky offered an overview of the state of PPC in Israel today. While there are only 80 pediatric oncology deaths per year, the emotional, social, and financial impact of these few patients is far-reaching. In a total of six major pediatric oncology centers in Israel, there are only three PPC doctors and two PPC trained nurse practitioners. In the community setting, there is only one PPC specialist, leaving most home care to be administered by practitioners untrained in PPC. Only one hospice is dedicated to pediatric oncology. PPC in Israel is uncomprehensive, fragmented geographically, understaffed, and underfunded.

Dr. Leeat Granek presented the impact of a child’s death on healthcare providers. Pediatric oncologists can develop deep emotional attachments to their patients, resulting in intense grief upon their deaths, including anticipatory grief, as well as a deep sense of failure. These feelings can have a long-lasting effect on both the personal and professional lives of pediatric oncologists [40]. There is a role for PPC providers in supporting the pediatric oncology teams by assisting with communication with parents and patients and administering medical, emotional, and spiritual care to patients, as well as supporting medical team staff with processing and coping with the emotional toll of their work.

Mrs. Lynne Dale-Halamish shared a few clinical stories illustrating how many children can speak clearly and openly about their impending death, surprisingly without apparent fear or psychological trauma. Two choices are available for communicating with a dying child: isolating the child by ignoring him/her or lying, or alternatively, to accompany the child on his/her final journey via good communication.

### The law - Israel’s dying patient act

Prof. Avraham Steinberg, who chaired the Knesset (Parliamentary) committee which prepared this legislation, detailed the ethical constructs and tradeoffs that went into the creation of this act [20]. They did their best to balance the sometimes conflicting values of sanctity of life and patient autonomy. He emphasized the Jewish values imbued in the act, in contrast with similar, yet more secularly-based legislation in other Western countries. Prof. Arie Ben-Yehuda described the difficulties in implementing the law in clinical practice (internal medicine). Difficulty in prognostication and time constraints on discussions with patients and families prior to critical events, both in in-patient settings and in the community, all play a role. Dr. Maya Peled-Raz presented the experience of the Bnei-Zion Medical Center in improving the clinical implementation of the Act. After exploring the ethical rationale for such legislation, the emotional and practical barriers to implementation were identified, and a local decision-making pathway was promoted by at least two EOL case managers in each department. A process for identification of EOL needs was adapted to the needs of each department – more proactive in specialized wards and more reactive in the intensive care and emergency departments. Staff training was provided and tools were created to promote implementation, including pop-up alerts on the electronic patient record flagging the existence of ADs and an easily accessible flow chart for decision-making.

### The role of government

**Dr. Irit Laxer** of the MoH provided insights into the National Program for Palliative Care, which began with a steering committee that assessed the PC capacity in Israel and made recommendations for improvement. These suggestions, published in 2016, included training health professionals at all levels, raising public awareness of advanced care planning, promoting standards and services in the hospital, community, and at-home settings; and promoting patient and family eligibility for services [22]. Mrs. Irit Fischer of the JDC-ESHEL, one of Israel’s foremost health and social service NGOs dedicated to the elderly [41], detailed progress made in implementing these recommendations. A JDC-ESHEL leadership training program has begun developing PC leadership teams in hospitals, nursing homes, and the community. Prof. Charles Sprung highlighted some of the difficulties facing the national program, including medical hesitation to prognosticate, strategies to increase the number of advance directives (AD), and the need for further PC training programs, as well as pressing areas for further research.

### The challenge of advance directives

The penultimate session was led by Prof. Dorith Shaham, along with Dr. Shelley Sternberg, Prof. Shai Lavi, and Mrs. Estelle Rubinstein. The speakers addressed the role of ADs in the face of family concerns and wishes, limited public awareness of the existence and utility of such directives, and the small number of completed documents currently deposited in the centralized database set up by the MoH. The timing and location of discussions regarding ADs also poses a challenge.

### Wrapping up

In the closing session, Dr. Iris Rasooly, a family physician, shared her personal perspective in caring for her ailing mother before she died. Dr. Sandy Buchman noted the dedication of the meeting participants to advancement of palliative care in Israel and expressed his appreciation for the progress that will be made by regulatory and medical institutions.

### Evaluation and lessons learned

The workshop on palliative care brought together a national representation of relevant stakeholders in Israel for the purpose of gaining a wider view of PC needs, impediments, and feasible solutions for moving forward. At the workshop, attendees had the opportunity to learn from parallel experience in the US, UK, and Canada, and assess it in the light of the Israeli setting. Participants highlighted the remarkable progress made by local initiatives in the face of minimal funding and a lack of central organization. The future direction of PC in Israel needs to include implementation of the national MoH plan, with particular attention to the following fundamental issues:

1. Appropriate funding and staffing for both PC inpatient and outpatient programs should be addressed. Without a sustainable financial model, the future of this service in Israel will remain fragile. PC has been shown to decrease health care expenditures both in Israel [42, 43] and abroad [44–46]. However, in the Israeli health care system, health care funds and hospitals often have competing and conflicting financial interests which hinder efforts to properly fund these programs.
2. In order to increase PC capacity, relevant education for primary caregivers and specialty PC training should be expanded. PC training should also be included in the curriculum of nursing and medical schools as well as internship, residency and fellowship programs. Core skills need to be included and standardized. Special emphasis should be put on training for nursing home staff including medical and nursing staff as well as caregivers.
3. Underserved populations including geriatric and psychiatric inpatients, minority populations, pediatric patients [47] and non-cancer patients should be addressed with education and funding opportunities.
4. Transfer of care from primary to specialty PC, from inpatient to outpatient or home PC should be seamless. To that end models of shared care and transition of care with integration of medical records should be built.
5. Public awareness of the importance and use of advance directives should continue to be promoted Initiatives such as “Five Wishes” (translated and promoted by the Clalit health fund), [48] “the Conversation project” (translated and promoted by the NGO Life’s Door) [49] and others, have helped to promote awareness of advance directives and national promotion of such projects should be encouraged. In addition, there is a need to increase the availability and convenience of completing and submitting advance directives in addition to improved access of medical teams as well as primary responders to these directives.
6. Regional and national social services as well as the national insurance should collaborate to create a comprehensive care package to support people dying at home and their families. This should include financial and social aid for primary caregivers tasked with caring for their loved ones at home. In addition – an interdepartmental protocol for dying at home, allowing for pronouncement of death in the home as a standard of care without the need to call an ambulance or open a police investigation – both of which are currently the norm.

In conclusion, there is a clear need for governmental support in the implementation of a national program described herein, including further meetings of relevant stakeholders to address the multiple legal, administrative, educational, cultural and clinical needs in building a sustainable and productive PC service accessible to all Israelis in need. The engagement of attendees was palpable, and their active participation demonstrated a commitment to the advancement of PC. The participants signed on to the ideal appearing in the name of the conference, namely that, while death is indeed inevitable, a bad death need not be.

## Data Availability

Not applicable.

## Appendix

Cases discussed at the meeting:

Clinical case # 1:

A 14 -year-old ultra-orthodox Jewish youth is dying from advanced resistant lymphoma. Throughout the course of his disease he has expressed no interest in any information about his disease and has deferred all decision making to his father. His mother has not been told about his dire condition although attempts have been made to explain the grave situation to the father.

The patient’s father insists that his son receive no opiates and that all measures should be taken to prolong his life including ventilation. As the patient begins to suffer from severe shortness of breath the team decides to intubate and begin mechanical ventilation. The team decides not to begin vasopressors or to transfer to ICU.

Questions:

- Would you consider ventilation in this setting to be life- prolonging, death extending or palliative care?
- Would you agree with the team’s decision regarding use of vasopressors?
- Would your decision change if the patient were admitted to an ICU?
- What if anything should we tell the patient?

Clinical case #2:

An 86-year-old cognitively intact woman with coronary artery disease and atrial fibrillation presents with a massive stroke. For 2 months, she remains unconscious with complete hemiplegia. Her chance of significant neurological recovery is virtually nil. She has no Advanced Directive. Her family has strong religious convictions and requests a permanent feeding tube. While this discussion occurs, she becomes febrile and is diagnosed with pneumonia.

The children insist that the doctors must under no circumstances tell the patient’s elderly husband her bleak prognosis, because “the information would surely kill him”. He is cognitively intact and keeps asking about his wife’s condition.

Questions:

- Is it appropriate to insert or refuse to insert a feeding tube?
- Should her pneumonia be treated?
- How should one respond to the husband’s queries?

## Abbreviations

AD: Advanced Directive
EOL: End-of-life
JDP: Joint Distribution Committee
MoH: Ministry of Health
NIHP: National Institute for Health Policy Research
PC: Palliative care
PPC: Pediatric palliative care
